# Use of structural equation models to predict dengue illness phenotype

**DOI:** 10.1371/journal.pntd.0006799

**Published:** 2018-10-01

**Authors:** Sangshin Park, Anon Srikiatkhachorn, Siripen Kalayanarooj, Louis Macareo, Sharone Green, Jennifer F. Friedman, Alan L. Rothman

**Affiliations:** 1 Center for International Health Research, Rhode Island Hospital, The Warren Alpert Medical School of Brown University, Providence, RI, United States of America; 2 Department of Pediatrics, The Warren Alpert Medical School of Brown University, Providence, RI, United States of America; 3 Institute for Immunology and Informatics, Department of Cell and Molecular Biology, University of Rhode Island, Providence, RI, United States of America; 4 Queen Sirikit National Institute of Child Health, Bangkok, Thailand; 5 Department of Virology, Armed Forces Research Institute of Medical Sciences, Bangkok, Thailand; 6 Division of Infectious Diseases and Immunology, Department of Medicine, University of Massachusetts Medical School, Worcester, MA, United States of America; Fundacao Oswaldo Cruz, BRAZIL

## Abstract

**Background:**

Early recognition of dengue, particularly patients at risk for plasma leakage, is important to clinical management. The objective of this study was to build predictive models for dengue, dengue hemorrhagic fever (DHF), and dengue shock syndrome (DSS) using structural equation modelling (SEM), a statistical method that evaluates mechanistic pathways.

**Methods/Findings:**

We performed SEM using data from 257 Thai children enrolled within 72 h of febrile illness onset, 156 with dengue and 101 with non-dengue febrile illnesses. Models for dengue, DHF, and DSS were developed based on data obtained three and one day(s) prior to fever resolution (fever days -3 and -1, respectively). Models were validated using data from 897 subjects who were not used for model development. Predictors for dengue and DSS included age, tourniquet test, aspartate aminotransferase, and white blood cell, % lymphocytes, and platelet counts. Predictors for DHF included age, aspartate aminotransferase, hematocrit, tourniquet test, and white blood cell and platelet counts. The models showed good predictive performances in the validation set, with area under the receiver operating characteristic curves (AUC) at fever day -3 of 0.84, 0.67, and 0.70 for prediction of dengue, DHF, and DSS, respectively. Predictive performance was comparable using data based on the timing relative to enrollment or illness onset, and improved closer to the critical phase (AUC 0.73 to 0.94, 0.61 to 0.93, and 0.70 to 0.96 for dengue, DHF, and DSS, respectively).

**Conclusions:**

Predictive models developed using SEM have potential use in guiding clinical management of suspected dengue prior to the critical phase of illness.

## Author summary

Dengue virus infection is one of the most critical public health issues, particularly in tropical and subtropical regions. This study developed statistical predictive models using the data obtained from 257 Thai children for dengue, dengue hemorrhagic fever, and dengue shock syndrome using structural equation modelling (SEM). We performed SEM based on clinical and laboratory factors on three and one day(s) prior to fever resolution. Our SEM models showed that age, tourniquet test, aspartate aminotransferase, and white blood cell, % lymphocytes, and platelet counts on three days prior to fever resolution were important risk factors for dengue and dengue hemorrhagic fever. Age, aspartate aminotransferase, hematocrit, tourniquet test, and white blood cell and platelet counts were important risk factors for dengue shock syndrome. Our predictive models showed good performances in the validation subjects (n = 897) who were not used for SEM, and thus we concluded that our predictive models can be practically used to guide clinical management of suspected dengue patients. Our study also showed that SEM can be used to predict the developments or severities of other illnesses.

## Introduction

Dengue virus (DENV) infection is a major public health issue worldwide particularly in tropical and subtropical regions. An estimated 390 million new DENV infections and 90 million cases of dengue illnesses are estimated to occur in more than 100 endemic countries, resulting in 20,000 deaths annually [1, 2]. In the past 50 years, the incidence of dengue has increased 30-fold [1, 3, 4].

DENV infection may result in a wide spectrum of disease severity ranging from asymptomatic infection to dengue fever (DF) and dengue hemorrhagic fever (DHF) [5]. DHF is characterized by fever, plasma leakage, bleeding diathesis, and thrombocytopenia, that in severe cases leads to shock (dengue shock syndrome, DSS) [5]. The mortality rate of DSS, up to 20% [6], is substantially reduced by timely replacement of intravascular fluid and blood losses, highlighting the importance of timely diagnosis of dengue, DHF, and DSS.

Several studies have developed diagnostic tools to predict the severity of an acute dengue illness [7–13]. Potts et al. developed predictive models using logistic regression analysis based on maximum or minimum levels of clinical laboratory variables during the illness [7] and classification and regression tree (CART) analysis based on clinical laboratory data on the day of presentation [8]. Chadwick et al. used logistic regression models based on clinical laboratory data within the first 2 days of presentation [9]. Brasier et al. used both logistic regression and a classification and regression tree analyses based on laboratory data on the day of presentation [10]. Recently, Nguyen et al. used logistic regression models based on laboratory data and nonstructural protein 1 rapid antigen testing on the day of presentation within ≤72 hours of fever [12]. These modeling approaches do not consider the underlying mechanisms of illness, are likely to overlook predictors for which opposite, indirect effects may offset one another, and cannot consider the relationship among covariates longitudinally. Predictive models which attempt to capture underlying mechanistic pathways might be a more biologically reliable and robust approach.

Progression of acute dengue illness is characterized by interdependent clinical and laboratory factors which change over the course of illness. Predictive models developed using structural equation model (SEM) have an advantage over models developed by general regression analysis because SEM can determine interdependent relationships among predictors and how they impact outcomes [14]. No studies have been performed to apply SEM approaches to predict dengue illness severity. The objective of this study was to construct statistical predictive models for dengue, DHF, and DSS by developing a series of SEMs.

## Methods

### Study setting

Data are from a longitudinal observational investigation at two hospitals in Thailand. Detailed descriptions of this investigation are provided elsewhere [7, 8]. Briefly, 1,384 children between 6 months and 15 years of age who presented with temperature of at least 38.5°C for less than 72 hours without any other identified source of infection were enrolled at Queen Sirikit National Institute of Child Health (QSNICH) in 1994–1997 (n = 506), 1999–2002 (n = 347), and 2004–2007 (n = 337) and the Kamphaeng Phet Provincial Hospital (KPPPH) in 1994–1997 (n = 194). Mean ages of each cohort were 8.9, 6.9, 7.8, and 8.9 years, respectively. In each cohort, 67.6% (DHF = 31.2%, DSS = 4.1%), 43.0% (DHF = 18.6%, DSS = 3.3%), 39.7% (DHF = 12.4%, DSS = 2.7%), and 61.4% (DHF = 18.3%, DSS = 3.8%) were diagnosed as having dengue, respectively. Most subjects were enrolled from the outpatient clinic. Each child was hospitalized per protocol and monitored in hospital until clinically stable and at least 1 day after defervescence. Children who had signs of shock at the first visit, chronic disease, or an initial alternate non-dengue diagnosis were excluded. All participants’ parents provided written informed consent prior to enrollment. This study followed the Ethical Principles for Medical Research Involving Human Subjects as defined by the Declaration of Helsinki and was approved by the Institutional Review Boards of the Ministry of Public Health (numbers: 102/2546, 71/2552, and 104/2552), Thailand, the US Army (numbers of Walter Reed Army Institute of Research: 436, 436b, and 1077/1620), and the University of Massachusetts Medical School (number: H-2222). Material has been reviewed by the Walter Reed Army Institute of Research. There is no objection to its presentation and/or publication. The opinions or assertions contained herein are the private views of the author, and are not to be construed as official, or as reflecting true views of the Department of the Army, the Department of Defense, or the National Institutes of Health. The investigators have adhered to the policies for protection of human subjects as prescribed in AR 70–25.

### Definitions

Fever day 0 was defined as the day of defervescence, when the temperature was less than 38°C for a consecutive 12 hours; days before and after defervescence were numbered consecutively. Study day 1 was defined as the day a child was enrolled in the study. Illness day 1 was defined as the day of onset of symptoms. Subjects (or their parent/guardian) were asked to identify the date of onset of their illness. This usually was the date of onset of fever.

Based on review of the medical records including study laboratory tests (see below), a physician who was not involved in patient care assigned a final clinical diagnosis as DF or DHF grade I to IV, guided by the 1997 World Health Organization guidelines [5].

### Laboratory testing

A venous blood specimen was collected daily. Plasma samples were tested for levels of aspartate aminotransferase (AST), alanine aminotransferase (ALT), and albumin using a Clinical System Analyzer (model 700; Beckmann Instruments, Brea, CA). Total white blood cell (WBC) count, platelet count, and hematocrit values were determined using a T540 hematologic analyzer (Coulter Electronics, Hialeah, FL). A tourniquet test was performed with the right and left arms alternately each day and the number of petechiae within a 1 sq in template (up to 20) was recorded; daily testing was stopped if the maximum value of 20 petechiae was recorded. Dengue was confirmed by viral isolation by mosquito inoculation and/or detection of viral RNA by reverse transcription polymerase chain reaction in plasma, and/or by serological assays (immunoglobulin M/G enzyme-linked immunosorbent assay and hemagglutination inhibition assay) of paired acute-convalescent plasma samples as described [15].

### Statistical analysis

**Fig 1:**
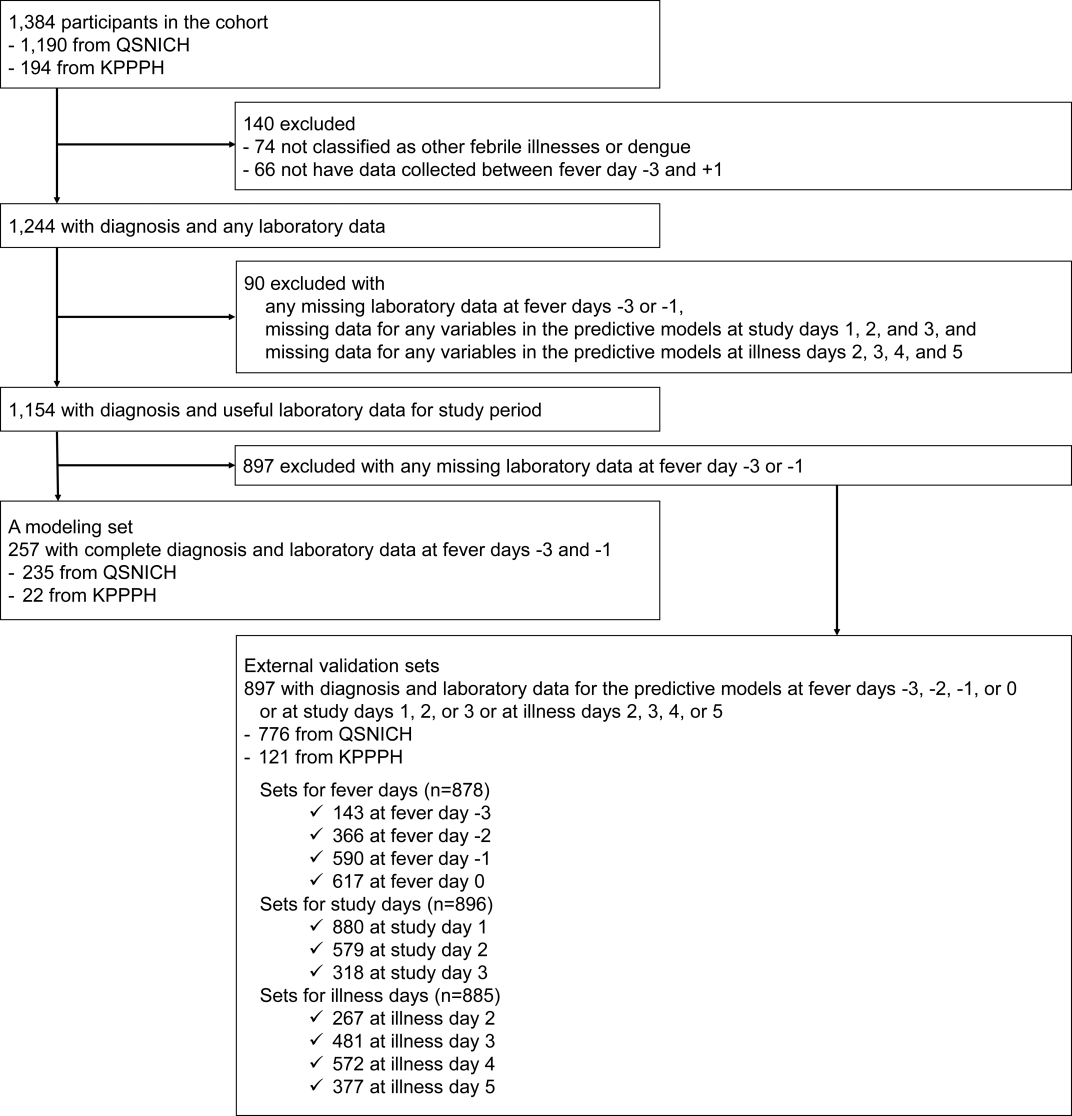
Study participants.

Our statistical analysis focused on the predictive value of clinical and laboratory data that are well validated for testing in typical clinical settings: tourniquet test, AST, ALT, albumin, WBC count, WBC differential, hematocrit, and platelet count. Outcomes of interest (dengue, DHF, or DSS) were defined as occurring at fever day +1, the final day of data collection and the day that a decubitus chest X-ray was performed to detect plasma leakage. To develop the predictive models, we used data from children who had all these variables obtained two and four days earlier (i.e., on fever days -3 and -1). There was a large amount of missing data on fever day -3 or -1 (totally 79.3%, ranging from 19.1% to 71.0% across variables) in 1,244 subjects who had a diagnosis of dengue or other non-dengue febrile illness and any laboratory data between fever day -3 and +1 (Fig 1). We therefore determined to perform complete analyses to build SEMs using the data from 257 subjects who had the complete data of predictors on fever day -3 or -1. To assess the sensitivity of our SEMs to missing data, we then employed multiple imputation for the full 1,244 subjects. We performed the Markov-chain Monte Carlo method to create 50 imputed data sets with no missing data. The results were pooled across the complete datasets.

**Fig 2:**
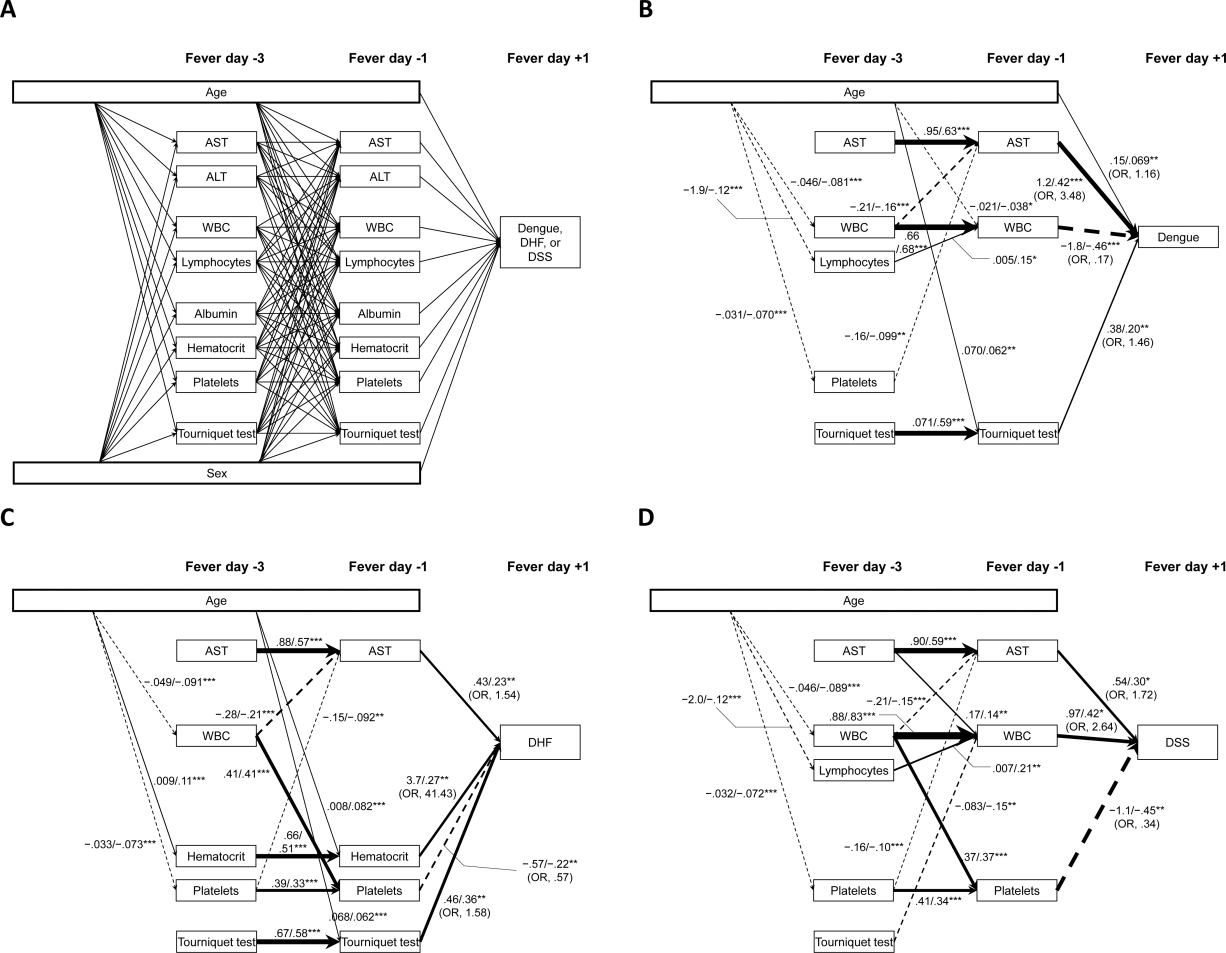
(A) Hypothesized pathways for developing dengue, DHF, or DSS and structural equation models predicting (B) dengue, (C) DHF, and (D) DSS. (B) The model fits were RMSEA = 0.057 and CFI = 0.979. (C) The model fits were RMSEA = 0.000 and CFI = 1.000. (D) The model fits were RMSEA = 0.095 and CFI = 0.937. Arrows indicate hypothetically significant positive or negative associations in (A). Solid and dotted arrows indicate significant positive and negative associations, respectively, in (B), (C), and (D). Unstandardized (= *B*) and standardized (= β) coefficients are shown next to the arrows. Correlations were omitted from the diagram. Unit: age, y; AST, U/mL; ALT, U/mL; WBC, cells/mm^3^; Lymphocytes, %; Albumin, g/dL; Hematocrit, %; Platelets, cells/mm^3^; Tourniquet test, petechiae/in^2^. AST, ALT, WBC, hematocrit, platelets, and tourniquet test were ln-transformed. Thickness of arrows was determined by standardized coefficients. *P value <0.05, **P value <0.01, ***P value <0.001.

SEMs were built using Mplus 8 statistical software (Muthén and Muthén 1998–2017). SEM parameters were estimated using the weighted least squares means and variances adjusted estimator with the theta parameterization. SEMs for dengue, DHF, and DSS were constructed based on the hypothesized mechanisms (Fig 2A). The minimum sample size for our SEMs was estimated as 207, together with degree of freedom of 66, significance level of 0.05, and desired statistical power of 0.80, on the basis of model-fitting with the root mean square error of approximation (RMSEA) [16, 17]. Our subjects for developing the predictive models exceeded the minimum required to achieve the desired power level. Significant predictors were retained in the final SEMs. AST, ALT, WBC count, hematocrit, platelet count, and tourniquet test were natural log-transformed after adding integer 1 to improve the distribution and homogeneity of variance. RMSEA below 0.07 and the comparative fit index (CFI) that exceed 0.95 indicate good model fit [18]. However, we did not use the criterion for the χ^2^ test, because the χ^2^ test is very sensitive to the large sample size [19]. In the sensitivity analyses using multiple imputation, we used the same criteria of RMSEA and CFI.

SEMs were used to develop the predictive models (see S1 Supporting Information). To achieve the earliest prediction, we used total effects of significant predictors at fever day -3 on the outcome of interest diagnosed at fever day +1. The performance of predictive models was examined by the receiver operating characteristic (ROC) curves and the area under the curves (AUCs) of the dataset acquired from 897 children who were not used to develop models in this study. The sensitivity (Se), specificity (Sp), positive predictive value (PPV), and negative predictive value (NPV) at the Youden index-based optimal cut-offs were calculated. Statistical analysis was performed using SAS 9.4 statistical software (SAS Institute, Cary, NC, USA), with exception of SEM. A *P* value < 0.05 was considered to be statistically significant. All data were anonymously analyzed.

## Results

Of the 257 children used to develop SEMs; 60.7% (n = 156), 19.8% (n = 51), and 3.5% (n = 9) of these children were diagnosed as dengue, DHF, and DSS, respectively. At fever days -3 and -1, AST, ALT, WBC count, hematocrit, platelet count, and tourniquet test were significantly different between groups (non-dengue, dengue but non-DHF, DHF but non-DSS, and DSS, Table 1). Pearson correlation coefficients among predictors and outcomes are presented in S2 Supporting Information. Children who were used to develop SEMs (n = 257) were not significantly different from those used for validation of SEM-based predictive models (n = 897) with respect to age (8.1 *vs*. 7.8 years), sex (48.3 *vs*. 43.6% girls), and the proportions of cases with DHF [17.3% (n = 155) for the validation set] and DSS [3.2% (n = 29) for the validation set] (*P* values of t-test or χ^2^ test >0.05). Moreover, the characteristics of 257 children used to develop SEMs were not significantly different from the 987 (= 1,244–257) children who had a diagnosis of dengue or non-dengue febrile illness and any laboratory data between fever day -3 and +1 but were not used to develop SEMs, in terms of age, sex, and the proportions of cases with DHF and DSS (*P* values of t-test or χ^2^ test >0.05). A higher percentage of subjects in the validation set were enrolled at KPPPH (8.6 *vs*. 13.5% for developing and validation sets, respectively; *P* value of χ^2^ test = 0.035).

**Table 1:** Characteristics of study participants (n = 257).

	Non-dengue	Dengue, non-DHF	DHF, non-DSS	DSS	*P* value
n	101 (39.3%)	105 (40.9%)	42 (16.3%)	9 (3.5%)	
Sex (= girl)	51 (50.5%)	51 (48.6%)	14 (33.3%)	8 (88.9%)	0.018
Age, yr	6.8 (6.2–7.3)	8.9 (8.4–9.5)^b^	9.3 (8.3–10.4)^b^	8.5 (6.1–10.9)	<0.001
At fever day -3					
AST, U/mL^a^	35.8 (33.4–38.3)	45.9 (42.0–50.3)^b^	47.7 (39.8–57.2)^b^	65.7 (33.5–128.1)^b^	<0.001
ALT, U/mL^a^	18.6 (17.2–20.1)	23.0 (20.6–25.7)^b^	23.3 (18.9–28.7)	31.7 (15.4–64.4)^b^	0.003
WBC, 1,000 cells/mm^3,a^	7.4 (6.7–8.2)	4.2 (3.8–4.5)^b^	4.0 (3.4–4.6)^b^	4.2 (2.9–6.1)^b^	<0.001
Lymphocytes, %	27.1 (23.5–30.7)	24.3 (21.6–27.0)	23.6 (17.9–29.4)	24.6 (9.3–39.8)	0.57
Albumin, g/dL	4.6 (4.6–4.7)	4.7 (4.6–4.8)	4.8 (4.6–4.9)	4.4 (4.1–4.8)	0.33
Hematocrit, %^a^	38.3 (37.7–38.8)	38.9 (38.3–39.5)	39.8 (38.8–40.8)^b^	37.4 (34.8–40.2)	0.031
Platelets, 1,000 cells/mm^3,a^	228 (212–244)	190 (176–206)^b^	167 (136–205)^b^	158 (114–219)	<0.001
Tourniquet test, petechiae/in^2,a^	4.6 (3.6–5.8)	6.4 (5.1–7.9)	9.9 (7.4–13.1)^b^	8.4 (4.9–13.9)	0.002
At fever day -1					
AST, U/mL^a^	37.6 (33.8–41.7)	70.2 (61.4–80.2)^b^	95.1 (76.4–118.3)^b,c^	137.5 (75.2–250.8)^b,c^	<0.001
ALT, U/mL^a^	19.1 (17.3–21.1)	33.5 (29.0–38.7)^b^	38.9 (30.5–49.4)^b^	60.6 (23.8–152.0)^b^	<0.001
WBC, 1,000 cells/mm^3,a^	5.3 (4.9–5.9)	2.7 (2.5–2.9)^b^	2.7 (2.4–3.1)^b^	4.3 (2.8–6.5)^c,d^	<0.001
Lymphocytes, %	38.7 (35.2–42.1)	38.7 (36.3–41.2)	33.5 (29.2–37.7)	35.9 (23.8–48.0)	0.23
Albumin, g/dL	4.4 (4.4–4.5)	4.4 (4.3–4.5)	4.4 (4.2–4.6)	4.0 (3.4–4.5)^b^	0.06
Hematocrit, %^a^	37.3 (36.6–38.1)	38.4 (37.7–39.2)	40.6 (39.3–41.8)^b,c^	42.0 (38.1–46.3)^b^	<0.001
Platelets, 1,000 cells/mm^3,a^	206 (190–223)	140 (128–153)^b^	110 (93–131)^b,c^	69 (40–118)^b,c,d^	<0.001
Tourniquet test, petechiae/in^2,a^	3.3 (2.4–4.3)	8.4 (6.7–10.5)^b^	12.2 (9.6–15.4)^b^	18.9 (16.2–22.1)^b^	<0.001

Data represent arithmetic or geometric^a^ mean (95% confidence interval) or n (proportion, %). ^b, c, and d^ Significantly different from non-dengue, dengue but non-DHF, and DHF but non-DSS, respectively, *P* value <0.05. *P* values were estimated by Fisher’s exact tests or analysis of variance followed by Bonferroni post hoc test.

The SEM for any dengue illness included age, AST, WBC count, % lymphocytes, platelet count, and tourniquet test at fever day -3 and AST, WBC count, and tourniquet test at fever day -1 (Fig 2B). AST, WBC count, and tourniquet test at fever day -3 were strongly predictive of their corresponding values at fever day -1. In addition, WBC and platelet counts were negative predictors of AST at fever day -1 and % lymphocytes was a positive predictor of WBC count at fever day -1. Taking both direct and indirect effects into account in SEM, the total effects of age, AST, and tourniquet test at fever day -3 were significantly related to an increased risk of any dengue illness, while WBC count, % lymphocytes, and platelet count were inversely related to risk of any dengue illness (Table 2).

**Table 2:** Total effect of predictors at fever day -3 in developing dengue, DHF, and DSS based on SEMs.

Outcome	Predictor	Unstandardized coefficient (= *B*)	Standardized coefficient (= β)	Odds ratio (95% confidence interval)	*P* value
Dengue					
	Age, y	0.298	0.140	1.35 (1.20–1.52)	<0.001
	AST, U/mL	1.191	0.265	3.29 (2.30–4.70)	<0.001
	WBC, cells/mm^3^	-1.422	-0.378	0.24 (0.14–0.42)	<0.001
	Lymphocytes, %	-0.009	-0.067	0.991 (0.983–0.999)	0.034
	Platelets, cells/mm^3^	-0.199	-0.042	0.82 (0.72–0.93)	0.002
	Tourniquet test, petechiae/in^2^	0.271	0.117	1.31 (1.07–1.60)	0.008
DHF					
	Age, y	0.110	0.079	1.12 (1.06–1.18)	<0.001
	AST, U/mL	0.380	0.129	1.46 (1.10–1.94)	0.008
	WBC, cells/mm^3^	-0.352	-0.135	0.70 (0.58–0.85)	<0.001
	Hematocrit, %	2.453	0.138	11.62 (2.11–64.08)	0.005
	Platelets, cells/mm^3^	-0.289	-0.092	0.75 (0.63–0.89)	0.001
	Tourniquet test, petechiae/in^2^	0.305	0.205	1.36 (1.10–1.67)	0.004
DSS					
	Age, y	-0.012	-0.009	0.99 (0.95–1.03)	0.52
	AST, U/mL	0.652	0.237	1.92 (1.07–3.44)	0.028
	WBC, cells/mm^3^	0.339	0.139	1.40 (0.80–2.45)	0.23
	Lymphocytes, %	0.007	0.087	1.007 (0.999–1.015)	0.11
	Platelets, cells/mm^3^	-0.527	-0.184	0.59 (0.42–0.83)	0.003
	Tourniquet test, petechiae/in^2^	-0.081	-0.063	0.92 (0.84–1.01)	0.08

Total effect was the sum of direct and indirect effects. AST, WBC, hematocrit, platelets, and tourniquet test were ln-transformed. AST, ALT, WBC, hematocrit, platelets, and tourniquet test were ln-transformed.

The SEM for DHF included age, AST, WBC count, hematocrit, platelet count, and tourniquet test at fever day -3 and AST, hematocrit, platelet count, and tourniquet test at fever day -1 (Fig 2C). AST, hematocrit, platelet count, and tourniquet test at fever day -3 were strongly correlated with their corresponding values at fever day -1. In addition, WBC count and platelet count were negative predictors of AST at fever day -1 and WBC count was a positive predictor of platelet count at fever day -1. Taking both direct and indirect effects into account, the total effects of age, AST, hematocrit, and tourniquet test at fever day -3 were related to increased risk of DHF at fever day +1, while WBC and platelet counts were inversely related to risk of DHF (Table 2).

The SEM for DSS included age, AST, WBC count, % lymphocytes, platelet count, and tourniquet test at fever day -3 and AST, WBC count, and platelet count at fever day -1 (Fig 2D). AST, WBC count, and platelet count at fever day -3 were strongly predictive of their corresponding values at fever day -1. In addition, WBC and platelet counts were negative predictors of AST at fever day -1, AST was a positive predictor of WBC count at fever day -1, and WBC count was a positive predictor of platelet count at fever day -1. Taking both direct and indirect effects into account, the total effect of AST at fever day -3 was positively significant in diagnosis of DSS at fever day +1, but that of platelet count was negatively significant (Table 2).

**Fig 3:**
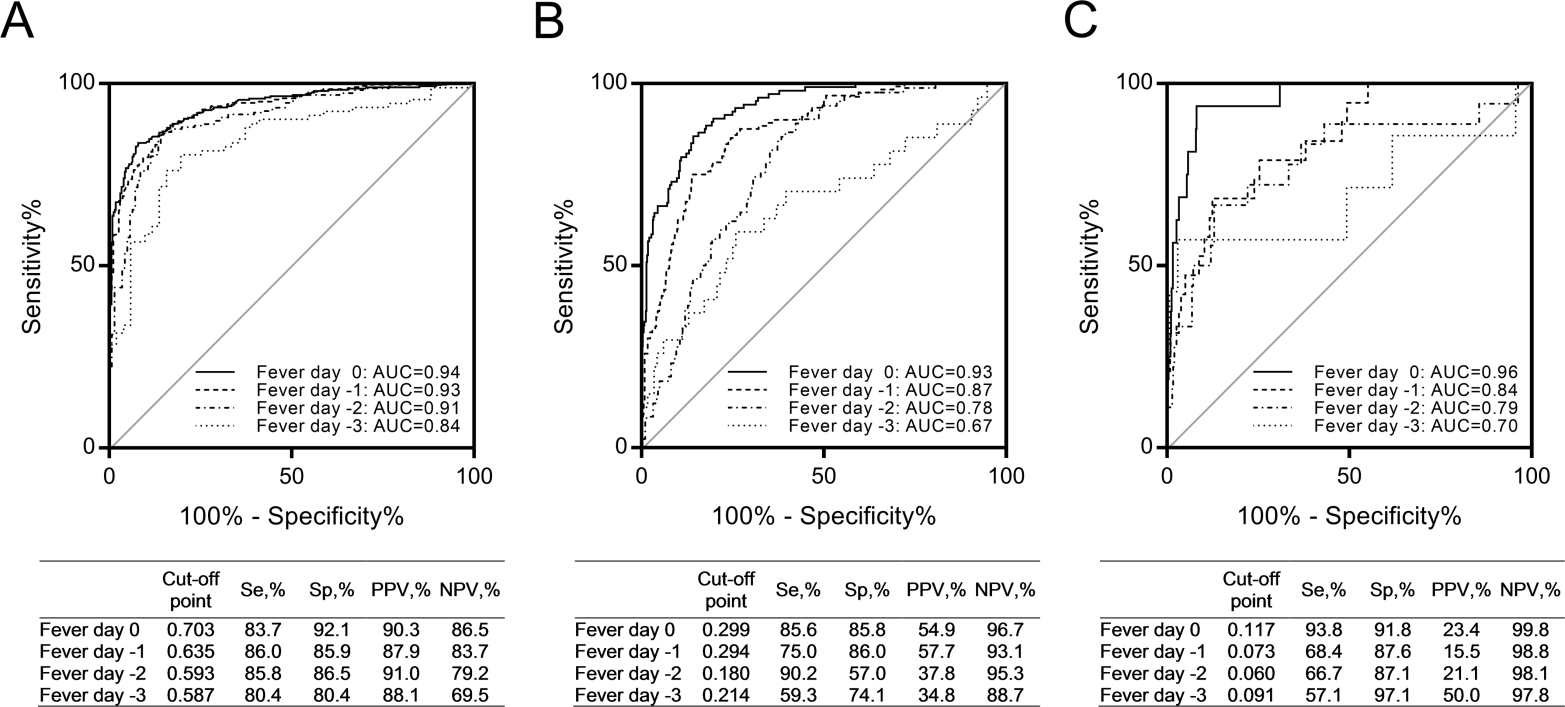
ROC curves for external validation employing fever days: (A) dengue, (B) DHF, and (C) DSS. n = 878 (n_fever day -3_ = 143, n_fever day -2_ = 366, n_fever day -1_ = 590, and n_fever day 0_ = 617).

For all three SEMs, fever day -1 parameters were predicted by fever day -3 parameters (see S3 Supporting Information). Therefore, we reasoned that data from a single time point might be sufficient for prediction of outcome. Using the regression coefficients for fever day -1 data, we derived revised SEM equations for dengue (*vs*. non-dengue illness), DHF (*vs*. all other diagnoses) and DSS (*vs*. all other diagnoses). The equations are available in S4 Supporting Information. The raw data used for SEM is available in S5 Supporting Information. We tested the predictive model over a range of fever days from -3 to 0; AUCs of the predictive model for dengue were in the range of 0.84 to 0.94 (Fig 3A). AUCs of the predictive model for DHF were in the range of 0.67 to 0.93 over fever days -3 to 0 (Fig 3B). Finally, AUCs of the predictive model for DSS were in the range of 0.70 to 0.96 over fever days -3 to 0 (Fig 3C). The proposed SEMs for dengue and DHF demonstrated good fit of the data, while the model for DSS demonstrated adequate fit of the data (Fig 2). In the sensitivity analyses using multiple imputations for missing values, our SEMs did not show good fits, but it was acceptable (RMSEA: 0.080 to 0.119, CFI: 0.896 to 0.953).

**Fig 4:**
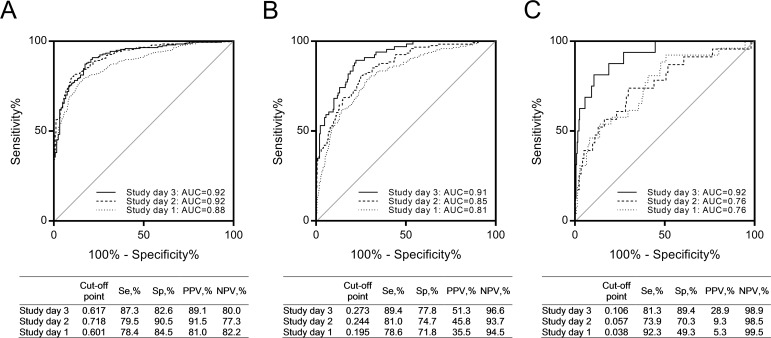
ROC curves for external validation employing study days: (A) dengue, (B) DHF, and (C) DSS. n = 896 (n_study day 1_ = 880, n_study day 2_ = 579, and n_study day 3_ = 318).

**Fig 5:**
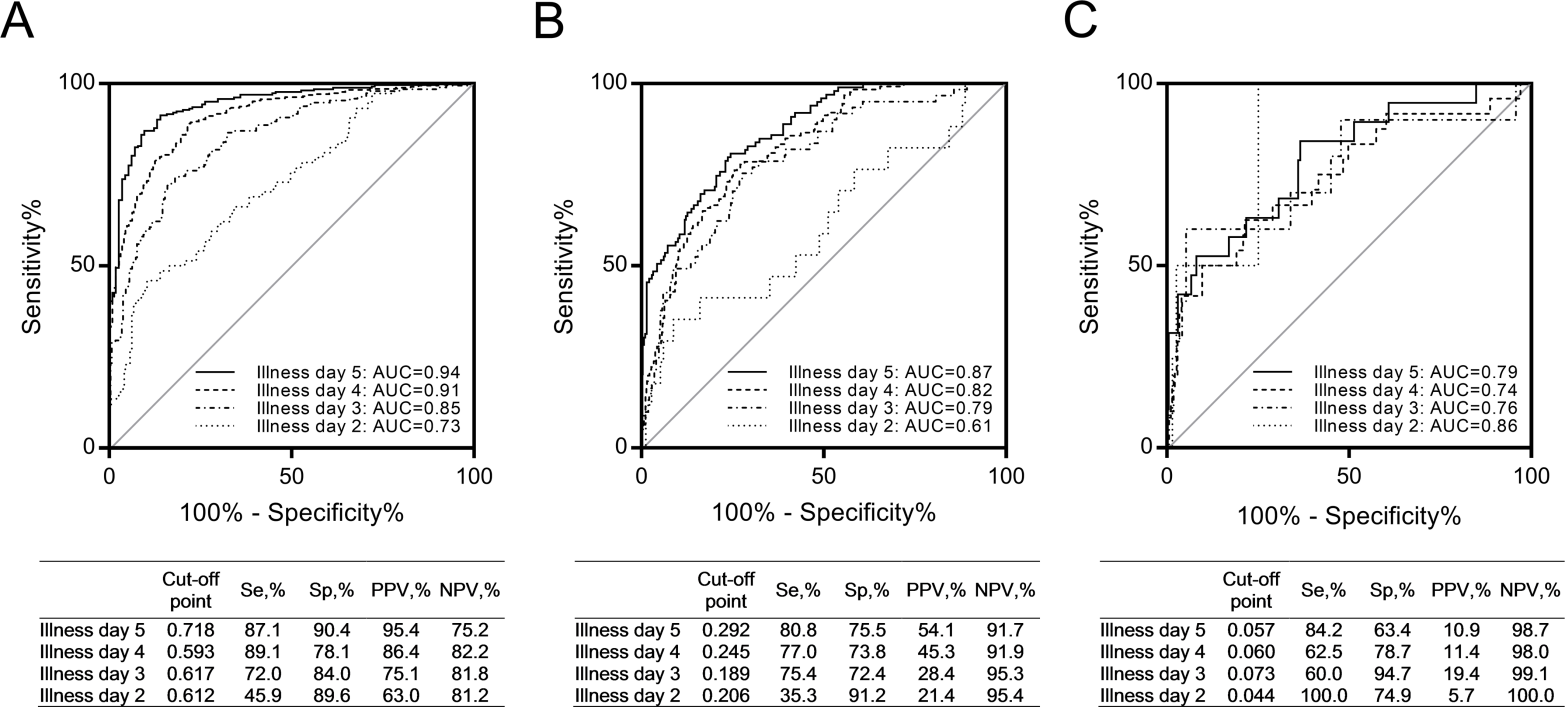
ROC curves for external validation employing illness days: (A) dengue, (B) DHF, and (C) DSS. n = 885 (n_illness day 2_ = 267, n_illness day 3_ = 481, n_illness day 4_ = 572, and n_illness day 5_ = 377).

Although the SEMs performed well over a range of fever day values, this parameter is defined retrospectively; therefore, we sought to further assess the performance of these models under typical clinical scenarios, by testing the models based on study day (time relative to presentation for medical care) and illness day (time relative to illness onset). AUCs of the predictive model for dengue were in the range of 0.88 to 0.92 and 0.73 to 0.94 for study days 1 to 3 (Fig 4A) and illness days 2 to 5 (Fig 5A), respectively. AUCs of the predictive model for DHF were in the range of 0.81 to 0.91 and 0.61 to 0.87 for study days 1 to 3 (Fig 4B) and illness days 2 to 5 (Fig 5B), respectively. AUCs of the predictive model for DSS were in the range of 0.76 to 0.92 and 0.74 to 0.86 for study days 1 to 3 (Fig 4C) and illness days 2 to 5 (Fig 5C), respectively.

## Discussion

We sought to develop predictive models to determine the probability of dengue, DHF, or DSS based on clinical and laboratory data available prior to the critical phase of illness which typically occurs within 24 hours of defervescence. We used SEMs to impose a hypothetical structure on the associations between these parameters. This approach allowed us to identify predictors at a single time point as early as fever day -3 which could predict the progression of disease. We further demonstrated that the SEM equations performed well using data from a wide range of time points during illness. The predictive accuracy of the SEMs improved using data collected at later time points, consistent with the progression of clinical and laboratory abnormalities in dengue.

Our SEMs identified elevated AST levels and decreased platelet count as significant predictors of all three outcomes of dengue, DHF, and DSS (Table 2). Previous studies observed that AST and ALT are commonly elevated in dengue and correlate with the severity of illness [15, 20, 21]. In our study, because these biomarkers were highly correlated (e.g., Pearson *r* at illness day -3 = 0.76), only AST remained in the SEMs. Our findings of an inverse relationship between platelet count and risk of dengue illness are also consistent with previous studies [7, 8, 12, 22, 23]. Decreased platelet count might be mechanistically linked to plasma leakage through its effects on vascular barrier function [24–26].

In the present study, older age, decreased WBC count, and increased number of petechiae in a tourniquet test were common significant risk factors for dengue and DHF. Although a meta-analysis reported an increased risk of DSS in younger children [23], which was attributed to their more permeable and fragile microvasculature [27], other studies reported contrasting findings [28]. These inconsistent results may be due to the differences in approaches to adjustment for potential confounders in statistical models. Indeed, several studies showed a significant association of severe dengue with age in univariable analyses but not in multivariable analyses [7, 12]. Although decreased WBC count was significantly associated with increased risks for dengue and DHF, this association was not significant for DSS. Both leukopenia and leukocytosis have been described in dengue, depending on the timing during illness [11, 29, 30]. Our findings for the tourniquet test were consistent with other studies which showed a crucial role for this clinical parameter in predictive models for acute dengue illnesses [7, 15, 31].

A novel aspect of SEM is the ability to identify correlations between factors contributing to prediction of the final outcome, which implies a mechanistic relationship. For example, all three models showed negative associations between WBC and platelet counts at fever day -3 and AST at fever day -1, and the models for DHF and DSS showed positive associations between WBC count at fever day -3 and platelet count at fever day -1. The positive association between WBC and platelet counts can potentially be explained by common mechanisms such as bone marrow suppression [30]. The negative association of WBC and platelet counts with AST is less clear, and may point to other mechanistic pathways such as immune activation [32].

Our predictive models showed good performance for identifying children who progressed to dengue, DHF, and DSS. We provided Se, Sp, PPV, and NPV at the Youden index-based optimal cut-offs, however, cut-off points may need to be modified depending on the objectives: for example, to increase Se for detecting a lethal condition. To capture all DSS cases using our SEM model with data from study day 3, it would be necessary to use a cut-off of 0.0172, with correspondingly lower Sp (55.3%) and PPV (10.6%) (Fig 5C).

Our study followed the case classification of DF, DHF, and DSS in use during the study period (and still in use in QSNICH and some other countries) rather than the alternative classification of dengue and severe dengue published in 2009 [33]. Our DSS cases correspond to severe dengue as defined in 2009, as there were no cases in our study with significant respiratory distress, clinically significant bleeding, or significant organ dysfunction. We did not collect data specifically for identification of cases as dengue with warning signs as defined in the 2009 classification.

To our knowledge, this is the first study to apply SEM to address the potential mechanisms as they evolve in acute dengue illnesses and to build predictive models based on clinical and laboratory data at various time points in the illness. Another strength of this study is that causality may be better ascertained through the prospective longitudinal study design. Further, our predictive models were developed based on easily obtained laboratory data and predictive performance of the models were evaluated over a variety of time criteria. Inclusion of biomarkers could further efforts to discern the mechanisms of disease. While we focused on acute dengue illnesses, our approach is likely to be generalizable to other acute illnesses.

Our study does have several limitations. First, in model development, selection bias might have arisen from the use of data on only 257 of 1,384 participants. Second, the model fit for DSS was borderline. DSS was a rare event (3.5%), and this likely resulted in instability of the SEM model. Third, PPVs in some DSS models were low, but this may also be attributed to the low prevalence of DSS [34]. Fourth, our predictive models were derived from Thai children, which may limit our ability to generalize these findings to patients in other areas.

In conclusion, our findings highlight the importance of AST and platelet count early in illness as indicators of dengue, DHF, and DSS. We also identified other early clinical indicators which can be used to predict outcomes. Our approach may serve as a methodological template to investigate the mechanisms of other illnesses using SEM.

## Supporting information

S1 Supporting Information Additional detail on statistical analysis. (DOCX)

S2 Supporting Information Pearson’s correlations. (DOCX)

S3 Supporting Information Intercepts and coefficients in SEMs. (DOCX)

S4 Supporting Information Additional detail on results. (DOCX)

S5 Supporting Information Data. (XLSX)

## References

[1] World Health Organization. Global strategy for dengue prevention and control 2012–2020. 2012.

[2] Bhatt S, Gething PW, Brady OJ, Messina JP, Farlow AW, Moyes CL, et al The global distribution and burden of dengue. Nature. 2013;496(7446):504–7. Epub 2013/04/09. 10.1038/nature12060 ; PubMed Central PMCID: PMCPMC3651993. 23563266 PMC3651993

[3] Shepard DS, Undurraga EA, Halasa YA, Stanaway JD. The global economic burden of dengue: a systematic analysis. Lancet Infect Dis. 2016;16(8):935–41. 10.1016/S1473-3099(16)00146-8 . 27091092

[4] Stanaway JD, Shepard DS, Undurraga EA, Halasa YA, Coffeng LE, Brady OJ, et al The global burden of dengue: an analysis from the Global Burden of Disease Study 2013. Lancet Infect Dis. 2016;16(6):712–23. 10.1016/S1473-3099(16)00026-8 ; PubMed Central PMCID: PMC5012511. 26874619 PMC5012511

[5] World Health Organization. Dengue haemorrhagic fever: diagnosis, treatment and control: World Health Organization; 1997 [1 November 2016]. Available from: http://www.who.int/csr/resources/publications/dengue/Denguepublication/en/.

[6] Guzman MG, Harris E. Dengue. Lancet. 2015;385(9966):453–65. 10.1016/S0140-6736(14)60572-9 . 25230594

[7] Potts JA, Thomas SJ, Srikiatkhachorn A, Supradish PO, Li W, Nisalak A, et al Classification of dengue illness based on readily available laboratory data. Am J Trop Med Hyg. 2010;83(4):781–8. 10.4269/ajtmh.2010.10-0135 ; PubMed Central PMCID: PMCPMC2946742. 20889865 PMC2946742

[8] Potts JA, Gibbons RV, Rothman AL, Srikiatkhachorn A, Thomas SJ, Supradish PO, et al Prediction of dengue disease severity among pediatric Thai patients using early clinical laboratory indicators. PLoS Negl Trop Dis. 2010;4(8):e769 10.1371/journal.pntd.0000769 ; PubMed Central PMCID: PMCPMC2914746. 20689812 PMC2914746

[9] Chadwick D, Arch B, Wilder-Smith A, Paton N. Distinguishing dengue fever from other infections on the basis of simple clinical and laboratory features: application of logistic regression analysis. J Clin Virol. 2006;35(2):147–53. 10.1016/j.jcv.2005.06.002 . 16055371

[10] Brasier AR, Ju H, Garcia J, Spratt HM, Victor SS, Forshey BM, et al A three-component biomarker panel for prediction of dengue hemorrhagic fever. Am J Trop Med Hyg. 2012;86(2):341–8. 10.4269/ajtmh.2012.11-0469 ; PubMed Central PMCID: PMCPMC3269290. 22302872 PMC3269290

[11] Lee IK, Liu JW, Chen YH, Chen YC, Tsai CY, Huang SY, et al Development of a Simple Clinical Risk Score for Early Prediction of Severe Dengue in Adult Patients. PloS one. 2016;11(5):e0154772 10.1371/journal.pone.0154772 ; PubMed Central PMCID: PMC4854400. 27138448 PMC4854400

[12] Nguyen MT, Ho TN, Nguyen VV, Nguyen TH, Ha MT, Ta VT, et al An evidence-based algorithm for early prognosis of severe dengue in the outpatient setting. Clin Infect Dis. 2017;64(5):656–63. 10.1093/cid/ciw863 . 28034883 PMC5850639

[13] Tanner L, Schreiber M, Low JG, Ong A, Tolfvenstam T, Lai YL, et al Decision tree algorithms predict the diagnosis and outcome of dengue fever in the early phase of illness. PLoS Negl Trop Dis. 2008;2(3):e196 10.1371/journal.pntd.0000196 ; PubMed Central PMCID: PMC2263124. 18335069 PMC2263124

[14] Bentler PM, Stein JA. Structural equation models in medical research. Stat Methods Med Res. 1992;1(2):159–81. Epub 1992/01/01. 10.1177/096228029200100203 . 1341656

[15] Kalayanarooj S, Vaughn DW, Nimmannitya S, Green S, Suntayakorn S, Kunentrasai N, et al Early clinical and laboratory indicators of acute dengue illness. J Infect Dis. 1997;176(2):313–21. . 9237695 10.1086/514047

[16] Kim KH. The relation among fit indexes, power, and sample size in structural equation modeling. Struct Equ Modeling. 2005;12(3):368–90. 10.1207/s15328007sem1203_2

[17] Gnambs T. Required sample size and power for SEM [9 June 2018]. Available from: http://timo.gnambs.at/en/scripts/powerforsem.

[18] Hooper D, Coughlan J, Mullen M. Structural equation modelling: Guidelines for determining model fit. EJBRM. 2008;6(1):53–60.

[19] Bentler PM, Bonett DG. Significance tests and goodness of fit in the analysis of covariance structures. Psychological bulletin. 1980;88(3):588.

[20] Kuo CH, Tai DI, Chang-Chien CS, Lan CK, Chiou SS, Liaw YF. Liver biochemical tests and dengue fever. Am J Trop Med Hyg. 1992;47(3):265–70. . 1355950 10.4269/ajtmh.1992.47.265

[21] Wang XJ, Wei HX, Jiang SC, He C, Xu XJ, Peng HJ. Evaluation of aminotransferase abnormality in dengue patients: A meta analysis. Acta tropica. 2016;156:130–6. 10.1016/j.actatropica.2015.12.013 . 26739659

[22] Lam PK, Ngoc TV, Thu Thuy TT, Hong Van NT, Nhu Thuy TT, Hoai Tam DT, et al The value of daily platelet counts for predicting dengue shock syndrome: Results from a prospective observational study of 2301 Vietnamese children with dengue. PLoS Negl Trop Dis. 2017;11(4):e0005498 10.1371/journal.pntd.0005498 . 28448490 PMC5407568

[23] Huy NT, Van Giang T, Thuy DH, Kikuchi M, Hien TT, Zamora J, et al Factors associated with dengue shock syndrome: a systematic review and meta-analysis. PLoS Negl Trop Dis. 2013;7(9):e2412 10.1371/journal.pntd.0002412 ; PubMed Central PMCID: PMCPMC3784477. 24086778 PMC3784477

[24] Aursnes I. Increased permeability of capillaries to protein during thrombocytopenia. An experimental study in the rabbit. Microvasc Res. 1974;7(3):283–95. . 4855533 10.1016/0026-2862(74)90016-8

[25] Kitchens CS, Weiss L. Ultrastructural changes of endothelium associated with thrombocytopenia. Blood. 1975;46(4):567–78. . 1174690

[26] Kitchens CS, Pendergast JF. Human thrombocytopenia is associated with structural abnormalities of the endothelium that are ameliorated by glucocorticosteroid administration. Blood. 1986;67(1):203–6. . 3940548

[27] Gamble J, Bethell D, Day NP, Loc PP, Phu NH, Gartside IB, et al Age-related changes in microvascular permeability: a significant factor in the susceptibility of children to shock? Clin Sci (Lond). 2000;98(2):211–6. . 10657278

[28] Lovera D, Martinez de Cuellar C, Araya S, Amarilla S, Gonzalez N, Aguiar C, et al Clinical Characteristics and Risk Factors of Dengue Shock Syndrome in Children. Pediatr Infect Dis J. 2016;35(12):1294–9. 10.1097/INF.0000000000001308 . 27455442

[29] Azin FR, Goncalves RP, Pitombeira MH, Lima DM, Branco IC. Dengue: profile of hematological and biochemical dynamics. Rev Bras Hematol Hemoter. 2012;34(1):36–41. 10.5581/1516-8484.20120012 ; PubMed Central PMCID: PMCPMC3459605. 23049382 PMC3459605

[30] La Russa VF, Innis BL. Mechanisms of dengue virus-induced bone marrow suppression. Baillieres Clin Haematol. 1995;8(1):249–70. . 7663049 10.1016/s0950-3536(05)80240-9

[31] Furlan NB, Tukasan C, Estofolete CF, Nogueira ML, da Silva NS. Low sensitivity of the tourniquet test for differential diagnosis of dengue: an analysis of 28,000 trials in patients. BMC Infect Dis. 2016;16(1):627 10.1186/s12879-016-1947-7 ; PubMed Central PMCID: PMC5095934. 27809813 PMC5095934

[32] Libraty DH, Endy TP, Houng HS, Green S, Kalayanarooj S, Suntayakorn S, et al Differing influences of virus burden and immune activation on disease severity in secondary dengue-3 virus infections. J Infect Dis. 2002;185(9):1213–21. Epub 2002/05/10. 10.1086/340365 . 12001037

[33] World Health Organization. Dengue guidelines for diagnosis, treatment, prevention and control: new edition 2009 [30 April 2017]. Available from: http://www.who.int/tdr/publications/documents/dengue-diagnosis.pdf. 23762963

[34] Dohoo I, Martin S, Stryhn H. Veterinary Epidemiologic Research: VER Inc; 2009.

