# Supplementary material for: Use of structural equation models to predict dengue illness phenotype
Source: PLoS Negl Trop Dis. 2018 Oct 1;12(10):e0006799. doi: 10.1371/journal.pntd.0006799 (PMC6181434; doi:10.1371/journal.pntd.0006799)
Supplement: S1 Supporting Information — (DOCX) [file pntd.0006799.s001.docx]

**METHODS**

**Statistical analysis**

Based on the measured levels of predictors at fever day -3, we calculated an expected value for the *m*th predictor of each patient at fever day -1 [Y*_m_*_(day -1)_´] by the following equation: Y*_m_*_(day -1)_´ = β_0(day -3)_ + β_1(day -3)_×X_1(day -3)_ + β_2(day -3)_×X_2(day -3)_ + … + β*_k_*_(day -3)_×X*_k_*_(day -3)_, where β_0(day -3)_ = the intercept and β_1(day -3),_ β_2(day -3), … ,_ and β_k(day -3)_ = unstandardized coefficients for measured variables X_1(day -3),_ X_2(day -3), … ,_ and X_k(day -3)_, respectively. The expected probability of each patient (P´) to be diagnosed with the outcome of interest at fever day +1 was given by the following formula: P´ = exp [logit (P´)]/{1 + exp [logit (P´)]}, where logit (P´) = β_0(day -1)_ + β_1(day -1)_×Y_1(day -1)_´ + β_2(day -1)_×Y_2(day -1)_´ + … + β*_m_*_(day -1)_×Y*_m_*_(day -1)_´, where β_0(day -1)_ = the intercept and β_1(day -1),_ β_2(day -1), … ,_ and β_m(day -1)_ = unstandardized coefficients for predicted variables Y_1(day -1)_´_,_ Y_2(day -1)_´_, … ,_ and Y_m(day -1)_´, respectively [1].

The expected probability for acute dengue illnesses of each child (P´) was recorded on a continuous scale (ranged from 0 to 1). Then, ROC curves, which are plotted by the sensitivity against the false positive rate (1- Sp) for a number of cut-off points of expected probability for acute dengue illnesses (P´), were produced for measured laboratory predictors at fever days -3, -2, -1, and 0, study days 1, 2, and 3, and illness days 2, 3, 4, and 5. AUCs were then quantified according to these ROC curves. The maximum Youden index [Se - (1 - Sp)] [2], was used to identify the optimum cut-off values from ROC curves to distinguish between children who would be diagnosed with acute dengue illnesses (dengue, DHF, or DSS) and non-illnesses.

**References**

1. Peng C-YJ, Lee KL, Ingersoll GM. An Introduction to Logistic Regression Analysis and Reporting. The Journal of Educational Research. 2002;96(1):3-14.

2. Youden WJ. Index for rating diagnostic tests. Cancer. 1950;3(1):32-5.
