# Supplementary material for: Use of structural equation models to predict dengue illness phenotype
Source: PLoS Negl Trop Dis. 2018 Oct 1;12(10):e0006799. doi: 10.1371/journal.pntd.0006799 (PMC6181434; doi:10.1371/journal.pntd.0006799)
Supplement: S2 Supporting Information — (DOCX) [file pntd.0006799.s002.docx]

**S2 Table.** Pearson’s correlations.

| Fever day | Variable |  |  |  | Day-3 | | | | | | |  | Day-1 | | | | | | | |  | Day +1 | | |
| --- | --- | --- | --- | --- | --- | --- | --- | --- | --- | --- | --- | --- | --- | --- | --- | --- | --- | --- | --- | --- | --- | --- | --- | --- |
|  |  | 2 |  | 3 | 4 | 5 | 6 | 7 | 8 | 9 | 10 |  | 11 | 12 | 13 | 14 | 15 | 16 | 17 | 18 |  | 19 | 20 | 21 |
|  | 1. Sex (=girl) | 0.02 |  | -0.08 | -0.06 | -0.03 | 0.08 | 0.09 | -0.05 | -0.09 | -0.07 |  | -0.1 | -0.04 | -0.04 | 0.01 | 0.06 | 0.00 | -0.03 | -0.01 |  | -0.04 | -0.05 | 0.15* |
|  | 2. Age, y |  |  | -0.08 | 0.07 | -0.25* | -0.36* | 0.07 | 0.34* | -0.21* | 0.10 |  | 0.11 | 0.21* | -0.34* | -0.27* | 0.06 | 0.43* | -0.23* | 0.20* |  | 0.35* | 0.17* | 0.02 |
| Day -3 | 3. AST, U/mL |  |  |  | 0.76* | -0.33* | 0.19* | -0.16* | -0.03 | -0.27* | 0.17* |  | 0.67* | 0.64* | -0.16* | 0.11 | -0.23* | -0.05 | -0.30* | 0.18* |  | 0.28* | 0.18* | 0.17* |
|  | 4. ALT, U/mL |  |  |  |  | -0.22* | 0.03 | -0.16* | 0.07 | -0.19* | 0.05 |  | 0.47* | 0.67* | -0.11 | -0.03 | -0.19* | 0.06 | -0.17* | 0.13* |  | 0.21* | 0.12* | 0.13* |
|  | 5. WBC, cells/mm^3^ |  |  |  |  |  | -0.37* | 0.08 | -0.10 | 0.42* | -0.28* |  | -0.45* | -0.39* | 0.69* | -0.22* | 0.12 | -0.13* | 0.48* | -0.25* |  | -0.52* | -0.23* | -0.07 |
|  | 6. Lymphocytes, % |  |  |  |  |  |  | -0.12 | -0.15* | -0.17* | 0.06 |  | 0.05 | 0.03 | -0.09 | 0.42* | -0.06 | -0.24* | -0.08 | -0.05 |  | -0.09 | -0.04 | -0.01 |
|  | 7. Albumin, g/dL |  |  |  |  |  |  |  | 0.16* | 0.16* | -0.02 |  | -0.19* | -0.21* | -0.09 | 0.00 | 0.53* | 0.04 | 0.06 | -0.07 |  | 0.05 | 0.03 | -0.09 |
|  | 8. Hematocrit, % |  |  |  |  |  |  |  |  | -0.19* | 0.08 |  | 0.03 | 0.10 | -0.11 | -0.17* | 0.01 | 0.60* | -0.13* | 0.09 |  | 0.12 | 0.10 | -0.08 |
|  | 9. Platelets, cells/mm^3^ |  |  |  |  |  |  |  |  |  | -0.08 |  | -0.35* | -0.32* | 0.25* | -0.05 | 0.24* | -0.16* | 0.49* | -0.11 |  | -0.25* | -0.20* | -0.10 |
|  | 10. Tourniquet test, petechiae/in^2^ |  |  |  |  |  |  |  |  |  |  |  | 0.23* | 0.16* | -0.18* | -0.02 | -0.11 | 0.05 | -0.18* | 0.51* |  | 0.20* | 0.20* | 0.05 |
| Day -1 | 11. AST, U/mL |  |  |  |  |  |  |  |  |  |  |  |  | 0.87* | -0.35* | 0.00 | -0.17* | 0.08 | -0.51* | 0.30* |  | 0.49* | 0.36* | 0.22* |
|  | 12. ALT, U/mL |  |  |  |  |  |  |  |  |  |  |  |  |  | -0.31* | -0.05 | -0.13* | 0.11 | -0.39* | 0.25* |  | 0.41* | 0.26* | 0.19* |
|  | 13. WBC, cells/mm^3^ |  |  |  |  |  |  |  |  |  |  |  |  |  |  | -0.18* | -0.01 | -0.16* | 0.42* | -0.24* |  | -0.58* | -0.17* | 0.06 |
|  | 14. Lymphocytes, % |  |  |  |  |  |  |  |  |  |  |  |  |  |  |  | 0.02 | -0.13* | -0.04 | -0.16* |  | -0.05 | -0.13* | -0.02 |
|  | 15. Albumin, g/dL |  |  |  |  |  |  |  |  |  |  |  |  |  |  |  |  | 0.01 | 0.31* | -0.10 |  | -0.07 | -0.08 | -0.16* |
|  | 16. Hematocrit, % |  |  |  |  |  |  |  |  |  |  |  |  |  |  |  |  |  | -0.30* | 0.15* |  | 0.22* | 0.28* | 0.16* |
|  | 17. Platelets, cells/mm^3^ |  |  |  |  |  |  |  |  |  |  |  |  |  |  |  |  |  |  | -0.26* |  | -0.44* | -0.38* | -0.28* |
|  | 18. Tourniquet test, petechiae/in^2^ |  |  |  |  |  |  |  |  |  |  |  |  |  |  |  |  |  |  |  |  | 0.41* | 0.29* | 0.17* |
| Day +1 | 19. Dengue (vs. other febrile illness) |  |  |  |  |  |  |  |  |  |  |  |  |  |  |  |  |  |  |  |  |  | 0.40* | 0.15* |
|  | 20. DHF (vs. all others) |  |  |  |  |  |  |  |  |  |  |  |  |  |  |  |  |  |  |  |  |  |  | 0.38* |
|  | 21. DSS (vs. all others) |  |  |  |  |  |  |  |  |  |  |  |  |  |  |  |  |  |  |  |  |  |  |  |

AST, ALT, WBC, hematocrit, platelet, and tourniquet test were ln-transformed. **P* value <0.05.
