# Supplementary material for: Use of structural equation models to predict dengue illness phenotype
Source: PLoS Negl Trop Dis. 2018 Oct 1;12(10):e0006799. doi: 10.1371/journal.pntd.0006799 (PMC6181434; doi:10.1371/journal.pntd.0006799)
Supplement: S3 Supporting Information — (DOCX) [file pntd.0006799.s003.docx]

**S3 Table.** Intercepts and coefficients in SEMs for developing dengue, DHF, and DSS

| Dengue | | | | | | | |  | DHF | | | | | | | |  | DSS | | | | | | | |
| --- | --- | --- | --- | --- | --- | --- | --- | --- | --- | --- | --- | --- | --- | --- | --- | --- | --- | --- | --- | --- | --- | --- | --- | --- | --- |
| *B* for dengue | |  | *B* for predictors at (-1) | |  | *B* for predictors at (-3) | |  | *B* for DHF | |  | *B* for predictors at (-1) | |  | *B* for predictors at (-3) | |  | *B* for DHF3/4 | |  | *B* for predictors at (-1) | |  | *B* for predictors at (-3) | |
| Variable | *B* |  | Variable | *B* |  | Variable | *B* |  | Variable | *B* |  | Variable | *B* |  | Variable | *B* |  | Variable | *B* |  | Variable | *B* |  | Variable | *B* |
| **Intercept** | **8.022** |  |  |  |  |  |  |  | **Intercept** | **-10.654** |  |  |  |  |  |  |  | **Intercept** | **-0.021** |  |  |  |  |  |  |
| **Age** | **0.147** |  |  |  |  |  |  |  | **AST*_(-1)_*** | **0.434** |  | **AST*_(-1)_*** |  |  |  |  |  | **AST*_(-1)_*** | **0.542** |  | **AST*_(-1)_*** |  |  |  |  |
| **AST*_(-1)_*** | **1.248** |  | **AST*_(-1)_*** |  |  |  |  |  |  |  |  | Intercept | 4.845 |  |  |  |  |  |  |  | Intercept | 4.186 |  |  |  |
|  |  |  | Intercept | 4.029 |  |  |  |  |  |  |  | AST*_(-3)_* | 0.876 |  |  |  |  |  |  |  | AST*_(-3)_* | 0.898 |  |  |  |
|  |  |  | AST*_(-3)_* | 0.954 |  |  |  |  |  |  |  | WBC*_(-3)_* | -0.278 |  | **WBC*_(-3)_*** |  |  |  |  |  | WBC*_(-3)_* | -0.205 |  | **WBC*_(-3)_*** |  |
|  |  |  | WBC*_(-3)_* | -0.207 |  | **WBC*_(-3)_*** |  |  |  |  |  |  |  |  | Intercept | 8.918 |  |  |  |  |  |  |  | Intercept | 9.165 |
|  |  |  |  |  |  | Intercept | 8.918 |  |  |  |  |  |  |  | Age | -0.049 |  |  |  |  |  |  |  | Age | -0.046 |
|  |  |  |  |  |  | Age | -0.046 |  |  |  |  | Platelets*_(-3)_* | -0.150 |  | **Platelets*_(-3)_*** |  |  |  |  |  | Platelets*_(-3)_* | -0.163 |  | **Platelets*_(-3)_*** |  |
|  |  |  | Platelets*_(-3)_* | -0.160 |  | **Platelets*_(-3)_*** |  |  |  |  |  |  |  |  | Intercept | 12.449 |  |  |  |  |  |  |  | Intercept | 12.498 |
|  |  |  |  |  |  | Intercept | 12.449 |  |  |  |  |  |  |  | Age | -0.033 |  |  |  |  |  |  |  | Age | -0.032 |
|  |  |  |  |  |  | Age | -0.031 |  | **Hct*_(-1)_*** | **3.724** |  | **Hct*_(-1)_*** |  |  |  |  |  | **WBC*_(-1)_*** | **0.972** |  | **WBC*_(-1)_*** |  |  |  |  |
| **WBC*_(-1)_*** | **-1.761** |  | **WBC*_(-1)_*** |  |  |  |  |  |  |  |  | Intercept | 1.181 |  |  |  |  |  |  |  | Intercept | -0.106 |  |  |  |
|  |  |  | Intercept | 2.584 |  |  |  |  |  |  |  | Age | 0.008 |  |  |  |  |  |  |  | AST*_(-3)_* | 0.170 |  |  |  |
|  |  |  | Age | -0.021 |  |  |  |  |  |  |  | Hct*_(-3)i_* | 0.659 |  | **Hct*_(-3)_*** |  |  |  |  |  | WBC*_(-3)_* | 0.876 |  | **WBC*_(-3)_*** |  |
|  |  |  | WBC*_(-3)_* | 0.660 |  | **WBC*_(-3)_*** |  |  |  |  |  |  |  |  | Intercept | 3.611 |  |  |  |  |  |  |  | Intercept | 9.165 |
|  |  |  |  |  |  | Intercept | 8.918 |  |  |  |  |  |  |  | Age | 0.009 |  |  |  |  |  |  |  | Age | -0.046 |
|  |  |  |  |  |  | Age | -0.046 |  | **Platelets*_(-1)_*** | **-0.568** |  | **Platelets*_(-1)_*** |  |  |  |  |  |  |  |  | Lymphocytes*_(-3)_* | 0.007 |  | **Lymphocytes*_(-3)_*** | |
|  |  |  | Lymphocytes*_(-3)_* | 0.005 |  | **Lymphocytes*_(-3)_*** | |  |  |  |  | Intercept | 3.728 |  |  |  |  |  |  |  |  |  |  | Intercept | 37.933 |
|  |  |  |  |  |  | Intercept | 40.860 |  |  |  |  | WBC*_(-3)_* | 0.406 |  | **WBC*_(-3)_*** |  |  |  |  |  |  |  |  | Age | -1.978 |
|  |  |  |  |  |  | Age | -1.910 |  |  |  |  |  |  |  | Intercept | 8.918 |  |  |  |  | Tourn*_(-3)_* | -0.083 |  |  |  |
| **Tourn*_(-1)_*** | **0.381** |  | **Tourn*_(-1)_*** |  |  |  |  |  |  |  |  |  |  |  | Age | -0.049 |  | **Platelets*_(-1)_*** | **-1.079** |  | **Platelets*_(-1)_*** |  |  |  |  |
|  |  |  | Intercept | 0.233 |  |  |  |  |  |  |  | Platelets*_(-3)_* | 0.394 |  | **Platelets*_(-3)_*** |  |  |  |  |  | Intercept | 3.918 |  |  |  |
|  |  |  | Age | 0.070 |  |  |  |  |  |  |  |  |  |  | Intercept | 12.449 |  |  |  |  | WBC*_(-3)_* | 0.372 |  | **WBC*_(-3)_*** |  |
|  |  |  | Tourn*_(-3)_* | 0.712 |  |  |  |  |  |  |  |  |  |  | Age | -0.033 |  |  |  |  |  |  |  | Intercept | 9.165 |
|  |  |  |  |  |  |  |  |  | **Tourn*_(-1)_*** | **0.456** |  | **Tourn*_(-1)_*** |  |  |  |  |  |  |  |  |  |  |  | Age | -0.046 |
|  |  |  |  |  |  |  |  |  |  |  |  | Intercept | 0.304 |  |  |  |  |  |  |  | Platelets*_(-3)_* | 0.406 |  | **Platelets*_(-3)_*** |  |
|  |  |  |  |  |  |  |  |  |  |  |  | Age | 0.068 |  |  |  |  |  |  |  |  |  |  | Intercept | 12.498 |
|  |  |  |  |  |  |  |  |  |  |  |  | Tourn*_(-3)_* | 0.670 |  |  |  |  |  |  |  |  |  |  | Age | -0.032 |
| Equations to predict blood levels in the fever day -1. | | | | | | | |  | Equations to predict blood levels in the fever day -1. | | | | | | | |  | Equations to predict blood levels in the fever day -1. | | | | | | | |
| **AST*_(-1)_´* = 4.029 + 0.954×X*_AST(-3)_* - 0.207×X*_WBC(-3)_* - 0.160×X*_Platelets(-3)_*** | | | | | | | |  | **AST*_(-1)_´* = 4.845 + 0.876×X*_AST(-3)_* - 0.278×X*_WBC(-3)_ -* 0.150×X*_Platelets(-3)_*** | | | | | | | |  | **AST*_(-1)_´* = 4.186 + 0.898×X*_AST(-3)_* - 0.205×X*_WBC(-3)_* - 0.163×X*_Platelets(-3)_*** | | | | | | | |
| **WBC*_(-1)_´* = 2.584 - 0.021×X*_Age_* + 0.660×X*_WBC(-3)_* + 0.005×X*_Lymphocytes(-3)_*** | | | | | | | |  | **Hct*_(-1)_´* = 1.181 - 0.008×X*_Age_* + 0.659×X *_Hct(-3)_*** | | | | | | | |  | **WBC*_(-1)_´* = -0.106 + 0.170×X*_AST(-3)_* + 0.876×X*_WBC(-3)_* + 0.007×X*_Lymphocytes(-3)_* - 0.083×X*_Tourn(-3)_*** | | | | | | | |
| **Tourn*_(-1)_´* = 0.233 + 0.070×X*_Age_* + 0.712×X*_Tourn(-3)_*** | | | | | | | |  | **Platelets*_(-1)_´* = 3.728 + 0.406×X *_WBC(-3)_* + 0.394×X*_Platelets(-3)_*** | | | | | | | |  | **Platelets*_(-1)_´* = 3.918+ 0.372×X*_WBC(-3)_* + 0.406×X*_Platelets(-3)_*** | | | | | | | |
| **Logit (P´) = 8.022 + 0.147×X*_Age_* + 1.248×X*_AST(-1)_´* - 1.761×X*_WBC(-1)_´* + 0.381×X*_Tourn(-1)_´*** | | | | | | | |  | **Tourn*_(-1)_´* = 0.304 + 0.068×X*_Age_* + 0.670×X*_Tourn(-3)_*** | | | | | | | |  | **Logit (P´) = -0.021 + 0.542×X*_AST(-1)_´* + 0.972×X*_WBC(-1)_´* - 1.079×X*_Platelets(-1)_´*** | | | | | | | |
|  | | | | | | | |  | **Logit (P´) = -10.654 + 0.434×X*_AST(-1)_´* + 3.724×X*_Hct(-1)_´* - 0.568×X*_Platelets(-1)_´* + 0.456×X*_Tourn(-1)_´*** | | | | | | | |  |  | | | | | | | |

Total effect was the sum of direct and indirect effects. Numbers in the parenthesis indicate the fever day. Expected probabilities for being diagnosed with the primary outcomes of each participant (P´) were calculated by the following equation: P´ = exp[Logit (P´)]/{1+exp[Logit (P´)]}. Unit: age, y; AST, U/mL; ALT, U/mL; WBC, cells/mm^3^; Lymphocytes, %; Albumin, g/dL; Hematocrit (Hct), %; Platelets, cells/mm^3^; Tourniquet test (Tourn), petechiae/in^2^. AST, WBC, Hct, platelets, and Tourn were ln-transformed.
