# Supplementary material for: Use of structural equation models to predict dengue illness phenotype
Source: PLoS Negl Trop Dis. 2018 Oct 1;12(10):e0006799. doi: 10.1371/journal.pntd.0006799 (PMC6181434; doi:10.1371/journal.pntd.0006799)
Supplement: S4 Supporting Information — (DOCX) [file pntd.0006799.s004.docx]

**RESULTS**

Using the regression coefficients for fever day -1 data, the SEM for dengue (*vs.* non-dengue illnesses) yielded the following equation: logit (P´) = 8.022 + 0.147×X*_Age_* + 1.248×(4.029 + 0.954×X*_AST(day -3)_* - 0.207×X*_WBC(day -3)_* - 0.160×X*_Platelets(day -3)_*) – 1.761×(2.584 - 0.021×X*_Age_* + 0.660×X*_WBC(day -3)_* + 0.005×X*_Lymphocytes(day -3)_*) + 0.381×(0.233 + 0.070×X*_Age_* + 0.712×X*_Tourniquet test(day -3)_*) (see S3 Supporting Information).

Similarly, the SEM for DHF (*vs.* all other diagnoses) yielded the following equation: logit (P´) = -10.654 + 0.434×(4.845 + 0.876×X*_AST(day -3)_* - 0.278×X*_WBC(day -3)_ -* 0.150×X*_Platelets(day -3)_*) + 3.724×(1.181 - 0.008×X*_Age_* + 0.659×X*_Hematocrits(day -3)_*) - 0.568×(3.728 + 0.406×X *_WBC(day -3)_* + 0.394×X*_Platelets(day -3)_*) + 0.456×(0.304 + 0.068×X*_Age_* + 0.670×X*_Tourniquet test(day -3)_*).

Finally, the SEM for DSS (*vs.* all other diagnoses) yielded the equation: logit (P´) = -0.021 + 0.542×(4.186 + 0.898×X*_AST(day -3)_* - 0.205×X*_WBC(day -3)_* - 0.163×X*_Platelets(day -3)_*) + 0.972×(-0.106 + 0.170×X*_AST(day -3)_* + 0.876×X*_WBC(day -3)_* + 0.007×X*_Lymphocytes (day -3)_* -0.083×X*_Tourniquet test(day -3)_*) - 1.079×(3.918+ 0.372×X*_WBC(day -3)_* + 0.406×X*_Platelets(day -3)_*).
